# Water-Jet Assisted Liposuction in Lipedema: Which Cannula is the Safest?

**DOI:** 10.1093/asjof/ojaf120

**Published:** 2025-09-26

**Authors:** Marie-Luise Aitzetmüller-Klietz, Jonah Berg, Tobias Hirsch, Matthias Aitzetmüller-Klietz

## Abstract

**Background:**

Liposuction in lipedema is a safe and effective treatment, but there currently are no studies analyzing the individual complications of water-jet-assisted liposuction in lipedema or the impact of the cannula's design.

**Objectives:**

The aim of the authors of this study is to answer the question which WAL cannula is the safest in lipedema patients and provide practitioners with the data they need to make an informed decision about the cannula they choose.

**Methods:**

The authors retrospectively analyzed complications and their underlying risk factors in 117 patients across 243 cases. Groups were formed by diameter (Ø) and number of ports of the used cannulas. Unpaired *t*-tests, Fisher's exact tests, and χ^2^ tests were used to analyze the patients’ characteristics for the complication rates across the cannulas.

**Results:**

Cannulas with 8 ports showed statistically significantly higher hemoglobin loss (*P* = .011), shorter incision-to-suture time (*P* = .023), and higher volume of aspirated fat (*P* < .001). The same results occurred when comparing the Ø3.8 mm cannulas that differ in the number of ports (4 vs 8 ports). The Ø4.8 mm group showed a significantly increased rate of wound-healing disorders compared with the Ø3.8 mm group (*P* = .041) and a statistically significantly higher aspirated fat volume (*P* = .014).

**Conclusions:**

No specific cannula showed superior safety in terms of complication rates. However, 8-port cannulas facilitated a faster aspiration of large volumes and reduced the incision-to-suture time compared with 4-port cannulas. This benefit was accompanied by a greater loss of hemoglobin. In contrast, cannula diameter played a less significant role in aspiration speed and did not increase the hemoglobin loss.

**Level of Evidence: 3 (Therapeutic):**

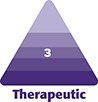

Lipedema is a chronic condition, initially described by Allen and Hines in 1940, leading to the deposition of disproportional volumes of subcutaneous fat in the lower extremities and/or arms.^1,2^

Lipedema is almost exclusively observed in women, with the estimated prevalence varying between 0.1% and 15%.^2-4^

Although familial accumulation and hereditary factors have been proven and hormonal trigger events such as puberty or pregnancy are described, the specific pathogenesis remains unclear.^1,5-8^

Guidelines for the definition and diagnosis criteria differ internationally. In this paper, we refer to the German guideline, which requires 2 major criteria for diagnosis: Firstly, a disproportionate fat accumulation of the extremities and secondly, that these affected regions produce painful sensations.^9^ Painful sensations include tenderness to palpation, spontaneous pain, and a sensation of heaviness in the affected areas.

In contrast to the consensus SOC guidelines for lipedema in the United States, the German S2k guideline requires painful sensations as mandatory diagnostic criteria: “Lipedema is always painful. A painless, disproportionate, and symmetrical disorder of fat distribution is referred to as lipohypertrophy […]”.^9,10^ Consequently, the German therapeutic approach defines symptom control of the painful sensations as the primary clinical objective.^9^

Therapeutic options are summarized in the German S1 guidelines for lipedema, including combined decongestive therapy, consisting of manual lymphatic drainage and the use of compressive garments and surgical options.^5^

If conservative therapy fails to provide adequate symptom control, liposuction can be performed.^9^ Liposuction remains the only option providing long-term symptom control, thereby significantly increasing the quality of life.^11,12^ This therapeutic approach is internationally established and supported by clinical studies.^12-14^

Different liposuction techniques are used, that is, ultrasound-assisted liposuction (UAL), laser-assisted liposuction, power-assisted liposuction (PAL), and water-jet-assisted liposuction (WAL) liposuction. Although the efficacy and application of most liposuction techniques have been explored extensively for aesthetic purposes, only WAL and PAL are recommended in lipedema patients.^9^ This paper focuses on WAL.

In most techniques, the tumescent fluid is injected into the adipose tissue before aspiration. In contrast, WAL utilizes cannulas that simultaneously inject tumescent fluid and aspirate fat. The cannula's tip contains a tiny nozzle that anatomizes the tumescent fluid into a water jet of tiny droplets, gently hydrodissecting the adipose tissue. Through a second channel inside the cannula, the fat is simultaneously aspirated.^15^

Scientific literature regarding lipedema is still very limited. Although the effectiveness of the WAL in patients with lipedema has been demonstrated, to our knowledge, there are no studies examining individual complications of WAL in lipedema.^16^

Cannulas are available in different lengths, shaft diameters (Ø), and number of ports at the cannula's tip. Although distributors advertise different cannula characteristics to be of advantage, there is neither accordance in users nor scientific evidence supporting these statements.

Shorter cannula shafts are thought to facilitate handling, but they necessitate more incisions when treating extensive fat deposits. Consequently, in lipedema, longer shafts may be advantageous, because the aspiration of large depots is required to achieve satisfactory outcomes. Considering Poiseuille's law, cannulas with larger shaft diameters should reduce resistance, thereby facilitating faster fat aspiration. Controversially, many surgeons believe that larger diameter cannulas cause greater tissue trauma, potentially leading to complications such as hematomas. Some surgeons even avoid using large cannulas altogether. The number of ports is also believed to influence the speed of aspiration, although some practitioners believe that multiport cannulas are unsafe and result in uneven outcomes because of the harsh aspiration. Other surgeons, however, prefer multiport cannulas because of the faster and more effortless aspiration.

Overall, there is insufficient evidence regarding the impact of the cannula's distinctive characteristics on the safety of the procedure, particularly regarding the WAL technique and/or patients with lipedema.

The objective of the authors of this study is to bridge this gap and provide surgeons with the necessary data to make informed decisions regarding the cannula they choose, thereby enhancing the safety of the liposuction in patients with lipedema.

## METHODS

### Study Design

This study is a retrospective cohort study analyzing the effect of different liposuction cannulas on adverse events in patients with lipedema. The patients’ cases were grouped by cannula diameter and number of ports in the cannula's tip.

### Participants

Patients were eligible if they met all the inclusion criteria and avoided all exclusion criteria.

All patients included were diagnosed with lipedema (ICD-10: E88.20-28) and received WAL at our department, a specialized center for lipedema treatment, between the beginning of April 2018 and the end of March 2023. Before liposuction a treatment plan was designed, and stage classification was conducted according to the German version of the internationally adopted 3-stage classification system.^5^ Patients were included if they met a minimum follow-up period of 3 months and if the cannulas’ reference numbers had been documented.

Exclusion criteria were missing reference numbers and liposuctions that were conducted for aesthetic reasons only (in patients with lipedema). Patients qualified for surgery if simultaneous manual lymphatic drainage and the wearing of compression garments for 6 months failed to provide adequate symptom control. All patients underwent examination by an anesthesiologist and were deemed suitable for surgery because all procedures were conducted in general anesthesia.

This study does not impose a BMI, stage, or age limit. However, liposuction procedures covered by health insurance had an upper BMI limit of 35 kg/m^2^ and required Stage II or III lipedema, as stipulated by the insurance terms. Patients who paid for the surgery without compensation from their health insurance were not subject to such BMI or stage limits, provided they were deemed suitable for general anesthesia.

Diabetics were advised to closely monitor their blood sugar levels in collaboration with their endocrinologist. Active smokers were informed about the detrimental effects of nicotine on wound healing and were encouraged to either pause or quit smoking.

The patients regularly presented for routine postoperative checkups. The intervals are tailored by the physician to the patients’ requirements, typically comprising 1 checkup during the initial postoperative week, 1 in the first month, and a long-term checkup after half a year. Standardized documentation of these checkups and of additional irregular checkups was used for data collection.

### Variables

Basic demographic data and disease characteristics were collected from the electronic health records (lipedema stage, age at surgery, weight before surgery, BMI before surgery, BMI by stages, diabetes mellitus, and smoker status).

To determine established complications of liposuction in body contouring surgeries, as well as general surgical complications, a literature review was conducted. The following complications were defined: perioperative fluid retention (see Discussion for details), surgical-site and/or systemic infection, necrosis of skin, blood transfusion, hematoma requiring additional treatment, secondary bleeding (ie, postoperative bleeding), wound-healing disorder of the incision site, uneven skin (ie, contour irregularities), edema of the lung, embolism, fat embolism, lymphedema, thrombosis, and newly occurred postoperative lymphedema.

Surgical parameters included incision-to-suture time, aspirated fat volume, and hemoglobin concentration preoperation and postoperation. Our department does not follow a standardized protocol for preoperative and postoperative laboratory testing; therefore, both the decision whether to perform laboratory testing and its timing were determined individually by the treating physicians. For this study, we included the last available preoperative and the first postoperative hemoglobin values, typically obtained 1 day before surgery and within the first 2 days following the procedure. We further calculated the mean hemoglobin difference per mean 1000 mL of aspirated fat and the mean incision-to-suture time per mean 1000 mL of aspirated fat.

The liposuction cannulas used were the WAL standard single-use cannulas by Human Med AG (Schwerin, Germany). Cannulas with the reference numbers 1500125 (Ø3.8 mm/L 30 cm/4 ports), 1501125 (Ø3.8 mm/L 30 cm/4 ports, RAPID sharp ports), 1503004 (Ø3.8 mm/L 30 cm/8 ports), 1503005 (Ø4.8 mm/L 30 cm/8 ports), and the infiltration Cannula 1500121 (Ø3.5 mm/L 30 cm/0 ports) were used in combination with the Body Jet Evo (Human Med AG).

All cannulas had the same length; therefore, only the cannulas’ diameter and the number of ports in the cannulas’ tip were included to group the patients. The cannulas’ reference numbers were extracted from the operation's usage documentation and traced back to the cannula's characteristic by Human Med.

Ethical approval for this study was granted by the Ethics Committee Westfalen-Lippe.

### Statistical Methods

In some procedures, >1 type of cannula was used. Cases were grouped by cannula characteristics, diameter, and number of ports.

Firstly, cases were grouped by the biggest cannula diameter used and by the cannula with the most ports.

Later, we excluded all operations that used >1 type of cannula to isolate the individual effect of the diameter and the ports on the complications (Supplemental Tables 1-10).

IBM SPSS Statistics (IBM SPSS Statistics, Armonk, NY), version 29.0.0.0, was used to perform all statistical calculations. A CI of 95% was chosen, with a double-sided *P*-value of <.05 being considered statistically significant.

To analyze the distribution of demographic data and disease characteristics among the groups, Fisher's exact test was used for binary variables, χ^2^ test for categorical variables with >2 features, and *t*-test for independent (ind.) samples for normally distributed data. Fisher's exact test was used to analyze the appearance of the individual complications in the different groups of cannula characteristics. *T*-test for independent samples was used to show the effect of the used cannula on incision-to-suture time and the aspirated fat volume.

All percentages stated refer to the total number of cases, not the number of patients.

### Treatment Protocol

All patients received liposuction at our department, a specialized center for lipedema treatment and liposuctions.

A treatment plan consisted of 4 individual liposuction sites: inner/ventral thigh, outer/posterior thigh, lower leg (circumferential), upper arm, and/or forearm. Patients only received liposuctions in regions that contribute to severe symptoms and therefore required medical attention. In most patients, multiple sites were symptomatic; therefore, patients requiring liposuction of different sites were treated in a staged manner to ensure sufficient healing time to increase patient safety. Individual treatment protocols were tailored to each patient.

Within our department, we generally did not aspirate >8% of the patient's body weight per session. However, we acknowledged that some patients might require a more radical approach in order to meet the patient's expectations. Therefore, the surgeon and the patient engaged in a collaborative discussion to determine the appropriate volume that aligns with the patient's expectation while simultaneously ensuring a safe liposuction. Liposuction sessions were scheduled at least 3 months apart to allow for adequate healing and recovery.

Because there currently exists no evidence for selection of WAL cannulas, surgeons chose the cannula based on personal preference. Our team consists of multiple surgeons—2 of whom specialize in lipedema. Although procedures were carried out by multiple surgeons, these 2 specialists were present and supervised all cases. All patients received a cefuroxime (1.5 g) single-shot antibiotic prophylaxis in the operating room preoperative, and every surgeon followed standard aseptic procedures meticulously. In WAL, infiltration and aspiration can be conducted simultaneously; therefore, most surgeons did not infiltrate the tumescent fluid before aspiration, contrary to how it is done in traditional tumescent liposuction. However, some surgeons chose to infiltrate a few minutes before aspiration, based on personal preference. The tumescent fluid contained 2 mg of epinephrine per 3000 mL 0.9% NaCl solution.

Patients usually stayed at the hospital for 1 to 3 nights, longer if medically required. Most patients received multiple liposuctions, resulting in a treatment period of multiple months to years. In this time, patients were seen regularly for postoperative checkups and preoperative planning, leading to an extensive, often multi-year follow-up time. Because of the overlapping follow-up periods resulting from multiple individual liposuction treatments, we were unable to accurately determine follow-up time for each individual treatment session. However, as per study inclusion criteria, all patients were confirmed to have a minimum follow-up duration of 3 months, which allowed for adequate assessment of immediate postoperative complications.

## RESULTS

### Participants

A total of 117 patients met inclusion criteria, undergoing 243 surgeries (=cases) in total. Patients’ demographical data and disease characteristics are shown in Table 1: 9 procedures were conducted in patients with Stage I lipedema (3.7%), 96 procedures with Stage II (39.5%), and 138 cases in Stage III (56.8%). The mean age on the day of surgery was 40.64 years (range, 19-70 years). The patients’ mean body weight was 93.7 kg (range, 62-159) with a mean BMI of 32.6 kg/m^2^ (range, 21.4-54.2 kg/m^2^). Twenty-three cases (9.5%) presented normal weight, 53 cases (21.8%) overweight, 94 cases (38.7%) obesity Class I, 46 cases (18.9%) obesity Class II, and 25 cases (10.3%) obesity Class III.

**Table 1.** Demographics and Disease Characteristics Cross All Cases

Number of cases	243
Lipedema stage, *n* (%)	
Stage I	9 (3.7)
Stage II	96 (39.5)
Stage III	138 (56.8)
Age, years	
Min	19
Average (SD)	40.6 (11.7)
Max	70
Weight, kg	
Min	62.0
Average (SD)	93.71 (18.89)
Max	159.0
Missing data, *n*	2
BMI, kg/m^2^	
Min	21.38
Average (SD)	32.60 (5.97)
Max	54.20
Missing data, *n*	2
BMI by stages, *n* (%)	
<18.5 kg/m^2^ (underweight)	0 (0)
18.5-24.9 kg/m^2^ (normal weight)	23 (9.5)
25.0-29.9 kg/m^2^ (overweight)	53 (21.8)
30.0-34.9 kg/m^2^ (obesity first class)	94 (38.7)
35.0-39.9 kg/m^2^ (obesity second class)	46 (18.9)
>40.0 kg/m^2^ (extreme obesity third class)	25 (10.3)
Diabetes mellitus, *n* (%)	9 (3.7)
Active smokers	44 (18.1)

Percentages relate to the number of cases, not the number of patients. SD, standard deviation.

Comorbidities included diabetes (3.7%) and smoking (18.1%). Given that lipedema is exclusively a female condition, all patients in the study cohort were female.

Supplemental Tables 1-4 show patients’ demographics broken down into the declared groups. No statistically significant differences were found between the groups.

### Outcome Data

In 192 cases, Cannula 1500125 or 1501125 (with identical dimensions: Ø3.8 mm/L 30 cm/4 ports); in 31 cases, Cannula 1503005 (Ø4.8 mm/L 30 cm/8 ports); in 33 cases, cannula 1503004 (Ø3.8 mm/L 30 cm/8 ports); and in 1 case, infiltration cannula 1500121 (Ø3.5 mm/L 30 cm/0 ports) were used.

In 70 (28.8%) cases, at least 1 complication occurred. Among those, perioperative fluid retentions were most prevalent with 55 cases (22.6%), followed by surgical-site and/or systemic infections with 19 cases (7.8%; Table 2). Rarely observed were necrosis of skin (*n* = 4, 1.6%), blood transfusions (*n* = 3, 1.2%), hematomas (*n* = 3, 1.2%), secondary bleedings (*n* = 3, 1.2%), wound-healing disorders (*n* = 3, 1.2%), and uneven skin (*n* = 2, 0.8%; Table 2). Edema of the lung, embolisms, fat embolisms, lymphedema, or thrombosis was not observed.

**Table 2.** Total Number of Complications and Complication Rates

	No. of cases (%)
Cases with 1 or more complication	70 (28.8)
Perioperative fluid retentions	55 (22.6)
Infections	19 (7.8)
Necrosis of skin	4 (1.6)
Blood transfusions	3 (1.2)
Hematomas	3 (1.2)
Secondary bleedings	3 (1.2)
Wound-healing disorders	3 (1.2)
Uneven skin	2 (0.8)

Percentages relate to the number of cases, not the number of patients.

The complication rates and procedure-related data for the different groups are given in Supplemental Tables 5-8. Preoperative and postoperative hemoglobin values were available for 68 cases (Table 3).

**Table 3.** Hemoglobin Difference, Incision-to-Suture Time, and Aspirated Fat Across All Cases

Preoperative and postoperative hemoglobin values	
Hemoglobin difference, g/dL	
Min	0
Average (SD)	−3.32 (1.70)
Max	−7.4
Missing data, *n*	175
Hemoglobin difference per 1000 mL of aspirated fat in g/dL/1000 mL	−0.8166
Incision-to-suture time, min	
Min	25
Average (SD)	81.3 (26.7)
Max	186
Missing data, *n*	4
Incision-to-suture time per 1000 mL aspirated in min/1000 mL of aspirated fat	19.9770
Aspirated fat, mL	
Min	100
Average (SD)	4068.7 (2103.6)
Max	11,400
Missing data, *n*	2

Percentages relate to the number of cases, not the number of patients. SD, standard deviation.

### Main Results

The Ø4.8 mm group showed a statistically significantly increased rate of wound-healing disorders in incision areas compared with the Ø3.8 mm group (6.7% vs 0.5%, *P* = .041) and a statistically significantly higher aspirated fat volume (average: 4953.1 vs 3943.3 mL, *P* = .014).

The rate of perioperative fluid retentions, infections, necrosis of skin, blood transfusions, hematomas, secondary bleeding, uneven skin, hemoglobin difference, and incision-to-suture time did not differ statistically significantly between the 2 groups (Table 4). The hemoglobin loss per liter of aspirated fat was similar between the cannulas (3.8 mm: −0.816 vs 4.8 mm: −0.810 g/dL/1000 mL), but the average aspiration time per liter of fat was shorter for the 4.8 mm cannulas (3.8 mm: 20.796 min/1000 mL vs 4.8 mm: 15.546 min/1000 mL).

**Table 4.** *P*-values of the Statistical Tests to Find Significant Differences Between the Cannulas

Complication or procedural variable	3.8 vs 4.8 mm cannula	4- vs 8-port cannula	3.8 mm cannula 4 vs 8 ports	8-port cannula 3.8 vs 4.8 mm
Perioperative fluid retentions	.641	.121	.067	.538
Infections	.482	.593	.155	.381
Necrosis of skin	1.000	1.000	.477	1.000
Blood transfusions	1.000	1.000	.384	No cases
Hematomas	1.000	.568	1.000	No cases
Secondary bleedings	1.000	.170	.058	.510
Wound-healing disorders	.041*	.170	1.000	.400
Uneven skin	.232	.458	No cases	.400
Aspirated fat volume	.014*	<.001*	<.001*	.588
Hb difference	.192	.011*	.016*	.472
Incision-to-suture time	.387	.023*	.001*	.517

*Statistically significant results.

**Figure 1.**
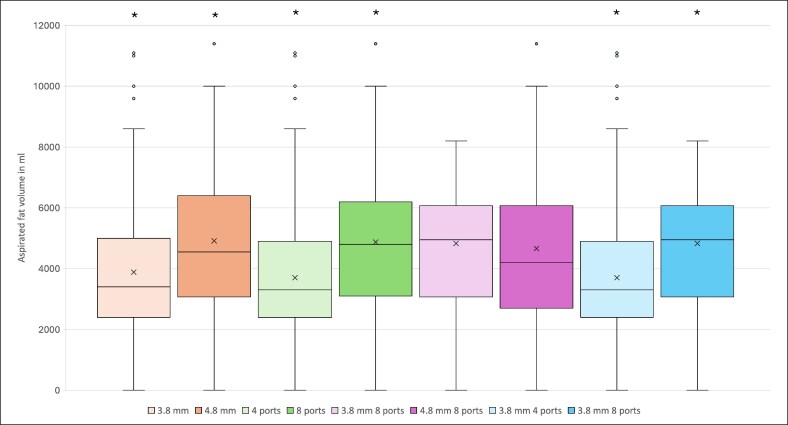
The image shows box plots, mean (x), and median (horizontal line) aspirated fat volume in milliliter for each cannula or cannula characteristic. The box represents the interquartile range. Dots outside of the whiskers are outliers. Significant differences are marked with an asterisk.

**Figure 2.**
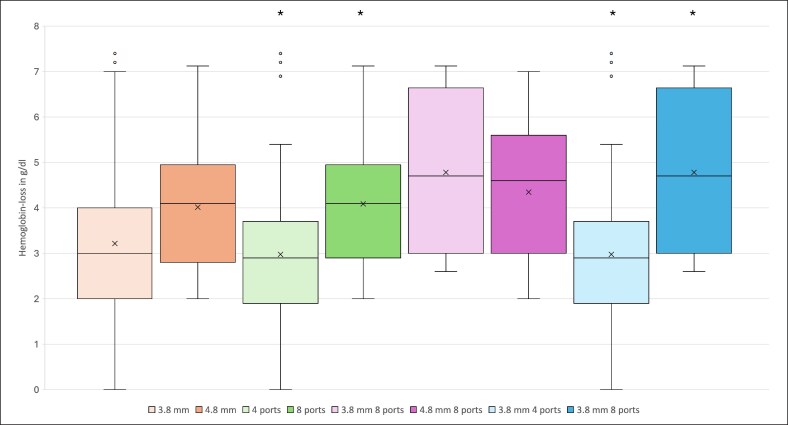
The image shows box plots, mean (x), and median (horizontal line) hemoglobin loss in g/dL for each cannula or cannula characteristic. The box represents the interquartile range. Dots outside of the whiskers are outliers. Significant differences are marked with an asterisk.

**Figure 3.**
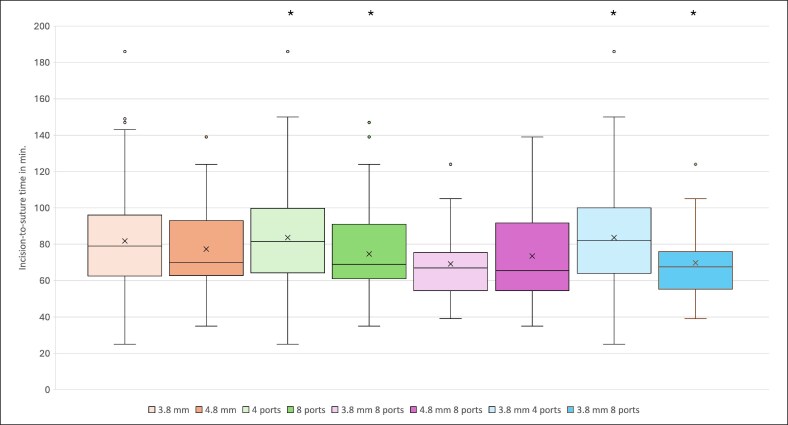
The image shows box plots, mean (x), and median (horizontal line) incision-to-suture time in minutes for each cannula or cannula characteristic. The box represents the interquartile range. Dots outside of the whiskers are outliers. Significant differences are marked with an asterisk.

Comparing cannulas with 4 ports against cannulas with 8 ports, 8-port cannulas showed statistically significantly higher hemoglobin loss (average: −4.09 vs −2.98 g/dL, *P* = .011), shorter incision-to-suture time (average: 75 vs 84 min, *P* = .023), and higher volume of aspirated fat (average: 5014 vs 3734 mL, *P* < .001; Figures 1-3). No statistically significant differences in the risk for any other outcome parameters were observed (Table 4). The 8-port cannulas showed a higher hemoglobin loss per liter of aspirated fat (−0.816 vs −0.798 g/dL/1000 mL) and a shorter average aspiration time per liter of fat compared with the 4-port cannulas (14.958 vs 22.496 min/1000 mL).

The same results occurred when comparing the Ø3.8 mm cannulas that differed in the number of ports (4 vs 8 ports). The Ø3.8 mm with 8 ports showed a statistically significantly higher hemoglobin loss (average: −4.35 vs −2.98 g/dL, *P* = .016), shorter incision-to-suture time (average: 70 vs 84 min, *P* = .001), and an elevated aspirated fat volume (average: 5247.0 vs 3738.0 mL, *P* < .001; Figures 1-3). All other outcome parameters did not differ statistically significantly (Table 4). The 3.8 mm 8-port cannulas showed a higher hemoglobin loss per liter of aspirated fat (−0.828 vs −0.797 g/dL/1000 mL) and a shorter average aspiration time per liter of fat compared with the 3.8 mm 4-port cannulas (13.341 vs 22.472 min/1000 mL).

When comparing cannulas with 8 ports that differed in diameter (3.8 vs 4.8 mm), no statistically significant differences regarding outcome parameters were observed. No cases of blood transfusion or hematomas were recorded in either group. No cases of secondary lymphedema were recorded in any group (Table 4, Supplemental Table 8). The 4.8 mm 8-port cannulas presented a higher hemoglobin loss per liter of aspirated fat (−0.981 vs −0.828 g/dL/1000 mL), and a slightly longer average aspiration time per liter of fat compared with the 3.8 mm 8-port cannula (15.179 vs 13.341 min/1000 mL).

## DISCUSSION

### Cannulas

To demonstrate the effect of different cannula characteristics, we compared cannula diameter and port count to the volume of aspirated fat. Because of the limited number of studies, comparison with other findings remains difficult.

Comparing the 2 different cannula diameters of 3.8 and 4.8 mm, a statistically significantly higher volume of fat was aspirated with 4.8 mm cannulas (Figure 1). This might give the impression of a relevant impact of cannula diameter on aspirated fat volume; however, it is important to clarify that only 4.8 mm cannulas with 8 ports were used.

When comparing all 4-port cannulas with 8-port cannulas, a statistically significantly higher volume of fat was aspirated with 8-port cannulas (Table 4, Figure 1).

The 3.8 mm 4-port cannulas and the 3.8 mm 8-port cannula were compared in relation to aspirated fat volume. The 3.8 mm cannulas with 8 ports aspirated statistically significantly more fat volume than those with 4 ports (Table 4, Figure 1).

On the other hand, there was no statistically significant difference between 3.8 mm 8-port cannulas and 4.8 mm 8-port cannulas regarding aspirated fat volume (Table 4, Figure 1). This leads us to the conclusion that the number of ports in a cannula had a higher impact on the aspirated fat volume than the diameter.

It has been demonstrated that multiport cannulas with bigger total port areas are faster at aspirating a given volume.^17^ Fodor et al also speculated that cannulas with more ports have more “active sites” and therefore aspiration of the fat is more efficient.^17^ This is in line with our findings. However, it is also important to mention that surgeons choose cannulas based on personal preferences. We suspect that surgeons rather chose 8-port cannulas when expecting large volumes of fat, because they believed them to be faster in aspiration. This presents a potential confounder and could also explain the correlation between higher number of cannula ports and larger aspirated fat volumes. This confounder could not be controlled because of the retrospective nature of this study.

Regarding the loss of hemoglobin concentration, we found a statistically significantly higher hemoglobin drop for 8-port cannulas compared with 4-port cannulas (Figure 2). This effect was also observed when comparing the 3.8 mm 4-port cannula with the 3.8 mm 8-port cannula. In contrast, no statistically significant difference in hemoglobin drop could be overserved comparing 3.8 and 4.8 mm cannulas and the 3.8 mm 8-port and the 4.8 mm 8-port cannulas (Table 4, Figure 2). This leads us to believe that the number of ports have a greater impact on blood loss than the cannula's diameter.

To account for the higher aspirated volume in the 8-port cannulas, we calculated the average hemoglobin loss per average liter of aspirated fat: although 8-port cannulas showed a statistically significantly higher volume of aspirated fat, they also showed a higher hemoglobin loss per liter of aspirated fat. The comparison between the 3.8 mm 4-port cannula and the 3.8 mm 8-port cannula showed even greater loss of hemoglobin per 1000 mL of aspirated fat in the 8-port group. In contrast, 3.8 and 4.8 mm cannulas presented very similar values (Supplemental Tables 5-8). We therefore suspect that a higher number of cannula ports may lead to a higher loss of hemoglobin per liter of aspirated fat, but we want to point out that this assumption is based on a limited number of cases.

We hypothesize that the cannulas glide through the tissue without causing damage to blood vessels because of their blunt tips, irrespective of their diameter. What does have an impact on rupturing small blood vessels are the cannulas’ ports, akin to the “teeth” of a cheese grater. A greater number of ports could correspond to a greater number of “teeth,” potentially leading to the rupture of more tiny blood vessels and consequently resulting in a higher hemoglobin loss.

Comparing the incision-to-suture time, 8-port cannulas showed a statistically significantly shorter procedure time compared with 4-port cannulas (Figure 3). This effect could be confirmed when comparing the 3.8 mm 4-port and 3.8 mm 8-port cannulas. On the other hand, the 3.8 mm cannula vs the 4.8 mm cannula and the 3.8 mm 8-port cannula vs the 4.8 mm 8-port cannula showed no significant difference in the incision-to-suture time (Table 4). Thus, the number of ports seems to have had a bigger impact on the incision-to-suture time.

Because a higher aspirated volume would logically increase the procedure's duration, we calculated the average minutes needed per average liter of aspirated fat and used this metric to determine the cannulas efficiency in aspirating large volume of fat: as expected, the 4-port cannulas required more time per aspirated liter compared with the 8-port cannulas, and the 3.8 mm 4-port cannula was also slower compared with the 3.8 mm 8-port cannula. We also observed that the 3.8 mm cannulas were slower than the 4.8 mm cannulas (Supplemental Tables 5-8). This seems to indicate that both the cannula's diameter and its number of ports are relevant for the incision-to-suture time. The incision-to-suture time is an important economical factor, especially in healthcare systems with high cost pressure.

### Complications

The rate of perioperative fluid retentions, infections, necrosis of skin, blood transfusions, hematomas, secondary bleeding, and uneven skin did not show statistically significant differences between the different cannulas, despite our large population (Table 4). Yet, we do want to report on the higher rates for perioperative fluid retention in 8-port cannulas vs the 4-port cannulas, the higher perioperative fluid retention and infection rate for the 3.8 mm 8-port cannula vs the 3.8 mm 4-port cannula, and the 4.8 mm 8-port cannulas vs the 3.8 mm 8-port cannula (Supplemental Tables 5-8). However, these observations did not reach statistical significance. Cannulas of 3.8 mm showed a significantly lower rate of wound-healing disorders compared with 4.8 mm cannulas but because of the very limited number of cases (1 vs 2), we consider this an incidental finding without clinical relevance.

### Surgical-Site Infections and Skin Necrosis

Surgical-site infections and sepsis in liposuctions are rare, but present the leading cause of deadly outcomes.^18^ Recent meta-analyses suggest an infection rate of 1% or lower across all liposuction techniques.^19,20^ We only found 2 studies that report on complications of WAL specifically, both not reporting any infections.^16,21^ However, both studies were not intentionally designed to detect potential complications and have a limited number of participants.

Our infection rate of 7.8% was elevated in comparison with literature. Our patients all received a cefuroxime single-shot antibiotic prophylaxis directly before surgery. Patients with a history of surgical-site infections also received an individual prophylactic antibiotic regiment in the first days postsurgery.

There is conflicting evidence concerning the use of prophylactic antibiotics in plastic surgery, with diverging recommendations for different procedures.^22-27^ There are currently no official recommendations regarding the use of antibiotics in liposuction.

BMI is a well-established risk factor for surgical-site infections in plastic surgeries and liposuctions in particular.^28,29^ Only 9.5% of our patients are of normal weight. The extremely elevated mean BMI of 32.6 kg/m^2^ might be responsible for the elevated infection rate in our study.

The elevated infection rate could also be attributed to the WAL or the lipedema itself; however, there is no sufficient data to verify these hypotheses.

The same applies to the risk of skin necrosis: multiple studies and meta-analyses report a rate of skin necrosis of 1% or lower for different liposuction techniques.^19,20,30,31^ We found skin necrosis in 4 patients (1.6%) and therefore consider the risk equal to other studies. Again, there are no studies researching skin necrosis in WAL liposuctions or in lipedema patients yet.

### Perioperative Fluid Retentions

In our study, we chose to use the descriptive-term perioperative fluid retention rather than seroma.

A major limitation in discussing seroma rates is that there is no universal definition of seroma. The pathogenesis of seromas is still unclear. A consensus consists in the fact that seromas seem to form as a consequence of dissecting large areas of tissue.

Seroma rates differ a lot between studies, ranging from 0% to 51%, with typically higher seroma rates for UAL, moderate rates for tumescent liposuctions, and low rates for dry liposuctions.^15,30-35^

Because we used WAL, we tried to compare with other studies using WAL, but existing data are limited.^15,36^ A study from 2011 reported no cases of seroma but included only 41 patients receiving WAL.^15^

There are different hypotheses about the etiology of the seroma fluid. Some studies suggest that a seroma consists of lymphatic fluid because of impaired lymphatic function/integrity, whereas others indicate an exudative process with an acute inflammatory genesis.^37^

These etiologies do not sufficiently explain our cases of perioperative fluid retentions.

Firstly, in our cohort, only 10 cases of the 55 cases with perioperative fluid retention showed any clinical sign of infection or inflammation in postoperative bloodwork.

Secondly, not a single patient developed a postoperative new lymphedema. This leads us to believe that the lymphatic function in our patients was not impaired. Therefore, we believe that impaired lymphatic function is not the cause of perioperative fluid retentions. Further, Stutz and Krahl conducted immunohistologic staining of the lipoaspirate in WAL and reported that the occurrence of lymphatic vessels in this aspirate is extremely rare in patients with lipedema.^38^

These 2 findings support our hypothesis that our perioperative fluid retentions are of a different pathogenesis than seromas.

We hypothesize these fluid retentions to be a unique complication of the WAL technique.

We believe that the high-pressure injection of the tumescent fluid into the tissue can lead to the formation of fluid-filled pockets. This might explain why our rate of perioperative fluid retention is higher than the seroma rate in studies on other liposuction techniques.

An analysis of the aspirate might explain its etiology. We recommend using the descriptive-term “perioperative fluid retention” instead of “seromas” to underline the unclear pathogenesis.

Regarding the clinical relevance of perioperative fluid retentions, some experts say that small seromas without progression in size should not be treated, as they often resorb without intervention.^39^ However, we experienced that patients benefit from the aspiration of even small fluid retentions, regarding pain and patient satisfaction. Thus, we increasingly used ultrasound to screen patients with postoperative pain for small fluid retentions.

This might lead to an attention bias in the presented rates. Because the implementation of the ultrasound screening nears the end of the study period, the rate of fluid retention clearly increased. Kuroi et al described that small Grade I seromas in mastectomy might often be overlooked with a resulting underestimated prevalence.^37^ We suspect the same effect for WAL, and that perioperative fluid retentions might be much more common in WAL than anticipated.

A prospective randomized controlled trial with and without aspiration of these fluid retentions would be needed to determine whether these fluid retentions would resorb without intervention and could answer the question of whether such fluid retentions should be considered a complication at all.

### Practical Implications

Given the retrospective nature of this study and the potential presence of numerous confounders, its findings should be interpreted and applied to clinical recommendations with caution. However, because this is the only study available on WAL in patients with lipedema and the safety of cannulas, we propose provisional recommendations for cannula selection.

Generally, all cannulas can be considered safe. Eight port cannulas were more efficient in aspirating large volumes of fat compared with 4-port cannulas, resulting in shorter incision-to-suture times, even when accounting for the increased total aspiration volume of the 8-port cannulas. This advantage comes with the disadvantage of a higher loss of hemoglobin, once again, even when adjusted for the higher total fat volumes. Interestingly, contrary to the common belief among surgeons, the diameter of the shaft was not as important to accelerate aspiration and did not impose the same disadvantage of a higher hemoglobin loss as the 8-port cannulas.

Therefore, we conclude that the selection of cannulas by surgeons should primarily focus on the number of ports rather than the shaft diameter. An ideal patient for the 8-port cannulas would have large estimated aspiration volumes and would otherwise be considered a low-risk patient. In contrast, patients with small fat deposits and an elevated risk profile should be treated with 4-port cannulas. The data do not support any recommendation regarding the shaft diameter; therefore, the diameter can be chosen by the surgeon's preference.

### Limitations

A major limitation of our study is the retrospective design. We heavily relied on the subjective free-form documentation of postoperative checkups with no standardized follow-up. For example, only 0.8% of uneven skin is not plausible, because most patients in Stage 3 lipedema experience uneven skin after liposuction. This is also the reason why we did not include subjective parameters like the aesthetic outcome in this study.

Another bias is the absence of randomization, which resulted in the surgeon selecting the cannulas. This could have led to the surgeon selecting 8-port cannulas in complex cases with larger fat deposits. Furthermore, the lack of randomization caused an imbalance in the used cannulas, with the 3.8 mm 4-port cannula being used in the majority of cases.

A prospective study would bring much more reliable data but is hard to realize because of the limited number of patients with lipedema that receive liposuction at a university hospital in Germany. Because our department did not routinely perform preoperative and postoperative blood tests for every patient, the number of cases with available hemoglobin levels was limited. The implementation of standardized protocols for perioperative blood testing could yield more robust and comprehensive data.

**Figure 4.**
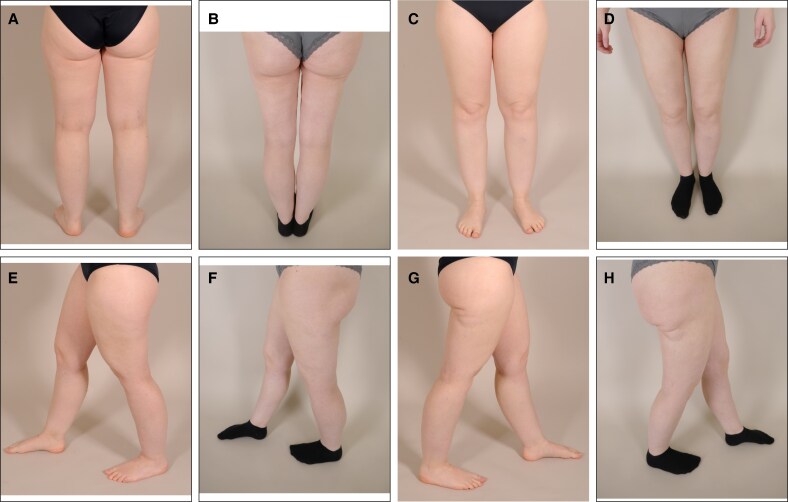
Patient 1: preoperative and postoperative clinical photographs of the lower extremities of a patient with Stage 1 lipedema. Age: 30 years, gender: female, preoperative BMI: 22.6 kg/m^2^. (A) Dorsal view of the legs—preoperative, (B) dorsal view of the legs—postoperative, (C) ventral view of the legs—preoperative, (D) ventral view of the legs—postoperative, (E) left lateral view of the legs—preoperative, (F) left lateral view of the legs—postoperative, (G) right lateral view of the legs—preoperative, (H) right lateral view of the legs—postoperative. Note the disproportionate adipose tissue distribution affecting the upper and lower legs preoperatively. The cuff sign—a characteristic abrupt transition between the lower leg and the foot—is clearly visible around the ankles in the preoperative images. The patient expressed particular concern about the fat accumulation in the lateral gluteal region; this area was deliberately included in the treatment plan to ensure a satisfactory outcome. The postoperative photographs were taken 3 years following the final procedure. The 3.8 mm 4-port cannulas were used for all procedures. Postoperatively, a volume reduction is clearly visible. The patient received an individual treatment protocol consisting of 3 liposuction sessions: (1) upper legs ventral/medial and lower legs (circumferential), (2) upper legs dorsal/lateral and lateral gluteal region, and (3) upper and lower arms.

**Figure 5.**
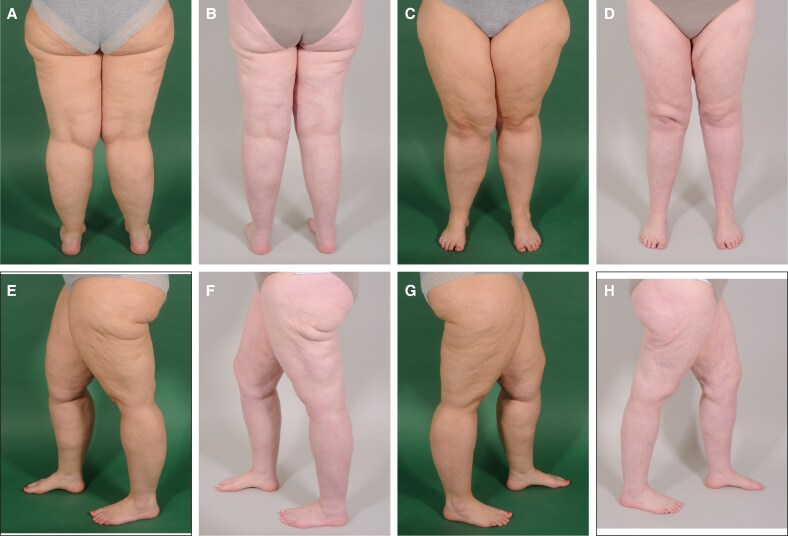
Preoperative and postoperative clinical photographs of the lower extremities of a patient with Stage 3 lipedema. Age: 32 years, gender: female, preoperative BMI: 34.2 kg/m^2^. (A) dorsal view of the legs—preoperative, (B) dorsal view of the legs—postoperative, (C) ventral view of the legs—preoperative, (D) ventral view of the legs—postoperative, (E) left lateral view of the legs—preoperative, (F) left lateral view of the legs—postoperative, (G) right lateral of the view legs—preoperative, (H) right lateral view of the legs—postoperative. Major fat depots are visible on the legs. The patient stated major symptoms in both legs. The postoperative photographs were taken 1 year and 3 months after the final procedure. In total, the patient received 5 liposuction sessions for the legs: (1) upper legs ventral/dorsal, (2) lower leg (circumferential), (3) upper legs dorsal/lateral, (4) upper legs dorsal/lateral, and (5) upper legs ventral/medial. This case demonstrates the challenges posed by stage 3 lipedema. Two sessions per region were needed to achieve adequate symptom control while simultaneously ensuring a safe procedure. Another challenge was skin laxity following liposuction. At a later stage, the patient subsequently underwent an upper leg lift to address this issue. For the first procedure on the upper legs, dorsal/lateral 4.8 mm 8-port cannulas were used. All other procedures were conducted using 3.8 mm 4-port cannulas.

Currently, there is no internationally consistent definition of lipedema. For instance, the German guideline specifies painful sensations in the affected regions as an obligatory diagnostic criterion, whereas the US consensus guideline regards pain merely as a possible symptom rather than an essential requirement. These inconsistencies may compromise the generalizability of studies involving lipedema patients. To mitigate this limitation, we provided preoperative and postoperative clinical images to bridge international differences in classification and enhance the global interpretability of our finding (Figures 4, 5). We consider our paper an important contribution to the international discourse on lipedema, given the existing differences in diagnostic and therapeutic approaches.

## CONCLUSIONS

No specific cannula design has demonstrated superior safety in terms of complication rates for the treatment of lipedema patients with WAL, and all cannulas appear equally safe in this regard. However, 8-port cannulas facilitated a faster aspiration of large volumes and reduced the incision-to-suture time compared with 4-port cannulas. This benefit was accompanied by a greater loss of hemoglobin. In contrast, cannula diameter played a less significant role in aspiration speed and did not increase the hemoglobin loss.

## Supplemental Material

This article contains supplemental material located online at https://doi.org/10.1093/asjof/ojaf120.

## Supplementary Material

ojaf120_Supplementary_Data
