# Supplementary material for: Water-Jet Assisted Liposuction in Lipedema: Which Cannula is the Safest?
Source: Aesthet Surg J Open Forum. 2025 Sep 26;7:ojaf120. doi: 10.1093/asjof/ojaf120 (PMC12596102; doi:10.1093/asjof/ojaf120)
Supplement: ojaf120_Supplementary_Data [file ojaf120_supplementary_data.zip › sup_Table 1_1.docx]

Supplemental table 1: Patient demographics and disease characteristics grouped by the biggest diameter cannula used in the procedures. Percentages relate to number of cases, not the number of patients.

|  |  | Ø 3.8mm | Ø 4.8mm | p-Value |
| --- | --- | --- | --- | --- |
| Number of Cases |  | 213 | 30 |  |
| Stage - No. (%) | Stage I | 9 (4.2) | 0 (0.0) | 0.446 |
|  | Stage II | 85 (39.9) | 11 (36.7) |  |
|  | Stage III | 119 (55.9) | 19 (63.3) |  |
| Age in Years | Min | 20 | 19 |  |
|  | Average (SD) | 41 (12) | 40 (13) | 0.944 |
|  | Max | 70 | 70 |  |
| Weight in kg | Min | 62 | 62 |  |
|  | Average (SD) | 93.8 (18.5) | 93 (21.6) | 0.828 |
|  | Max | 159 | 147 |  |
|  | Missing Data - No. | 2 | 0 |  |
| BMI in kg/m2 | Min | 21.38 | 22.14 |  |
|  | Average (SD) | 32.60 (5.98) | 32.57 (5.98) | 0.974 |
|  | Max | 54.20 | 49.12 |  |
|  | Missing Data - No. | 2 | 0 |  |
| BMI by Stages - No. (%) | < 18.5 kg/m^2^ (Underweight) | 0 (0) | 0 (0) | 0.821 |
|  | 18.5-24.9 kg/m^2^ (Normal Weight) | 19 (9.0) | 4 (13.3) |  |
|  | 25.0-29.9 kg/m^2^ (Overweight) | 48 (22.7) | 5 (16.7) |  |
|  | 30.0- 34.9 kg/m^2^ (Obesity 1st Class) | 81 (38.4) | 13 (43.3) |  |
|  | 35.0-39.9 kg/m^2^ (Obesity 2nd Class) | 40 (19.0) | 6 (20.0) |  |
|  | > 40.0 kg/m^2^ (Extreme Obesity 3rd Class) | 23 (10.9) | 2 (6.7) |  |
| Diabetes Mellitus – No. (%) |  | 7 (3.3) | 2 (6.7) | 0.307 |
| Active Smokers |  | 38 (17.8) | 6 (20.0) | 0.801 |
