# Supplementary material for: Water-Jet Assisted Liposuction in Lipedema: Which Cannula is the Safest?
Source: Aesthet Surg J Open Forum. 2025 Sep 26;7:ojaf120. doi: 10.1093/asjof/ojaf120 (PMC12596102; doi:10.1093/asjof/ojaf120)
Supplement: ojaf120_Supplementary_Data [file ojaf120_supplementary_data.zip › sup_Table 10_short_1_c.docx]

Supplemental table 10: Statistical test to find significant differences between the individual cannulas.

| Group 1 | Group 2 | Complication | Odds Ratio/Mean Difference | 95% Confidence Interval | p-Value |
| --- | --- | --- | --- | --- | --- |
| Ø3.8mm  4 Ports | Ø3.8mm  8 Ports | Perioperative Fluid Retentions | 2.169 | 0.954 – 4.934 | 0.067 |
| Ø3.8mm  4 Ports | Ø3.8mm  8 Ports | Infections | 2.441 | 0.803 – 7.415 | 0.155 |
| Ø3.8mm  4 Ports | Ø3.8mm  8 Ports | Necrosis of Skin | 1.944 | 0.196 – 19.318 | 0.477 |
| Ø3.8mm  4 Ports | Ø3.8mm  8 Ports | Blood Transfusions | 2.933 | 0.258 – 33.369 | 0.384 |
| Ø3.8mm  4 Ports | Ø3.8mm  8 Ports | Hematomas | - | - | 1.000 |
| Ø3.8mm  4 Ports | Ø3.8mm  8 Ports | Secondary Bleedings | 12.207 | 1.072 – 138.984 | 0.058 |
| Ø3.8mm  4 Ports | Ø3.8mm  8 Ports | Wound Healing Disorders | - | - | 1.000 |
| Ø3.8mm  4 Ports | Ø3.8mm  8 Ports | Uneven Skin | -1.508.814 | -2265.135 –  -752.492 | **< 0.001** |
| Ø3.8mm  4 Ports | Ø3.8mm  8 Ports | Aspirated Fat Volume | - | - | No Cases |
| Ø3.8mm  4 Ports | Ø3.8mm  8 Ports | Hb-Difference | 1.367 | 0.267 – 2.466 | **0.016** |
| Ø3.8mm  4 Ports | Ø3.8mm  8 Ports | Incision-To-Suture Time | 13.952 | 5.881 – 22.024 | **0.001** |
| Ø3.8mm  8 Ports | Ø4.8mm  8 Ports | Perioperative Fluid Retentions | 0.576 | 0.164 – 2.020 | 0.538 |
| Ø3.8mm  8 Ports | Ø4.8mm  8 Ports | Infections | 0.263 | 0.028 – 2.443 | 0.381 |
| Ø3.8mm  8 Ports | Ø4.8mm  8 Ports | Necrosis of Skin | - | - | 1.000 |
| Ø3.8mm  8 Ports | Ø4.8mm  8 Ports | Blood Transfusions | - | - | No Cases |
| Ø3.8mm  8 Ports | Ø4.8mm  8 Ports | Hematomas | - | - | No Cases |
| Ø3.8mm  8 Ports | Ø4.8mm  8 Ports | Secondary Bleedings | - | - | 0.510 |
| Ø3.8mm  8 Ports | Ø4.8mm  8 Ports | Wound Healing Disorders | - | - | 0.400 |
| Ø3.8mm  8 Ports | Ø4.8mm  8 Ports | Uneven Skin | 400.862 | 904.438 –  1706.162 | 0.588 |
| Ø3.8mm  8 Ports | Ø4.8mm  8 Ports | Aspirated Fat Volume | - | - | 0.400 |
| Ø3.8mm  8 Ports | Ø4.8mm  8 Ports | Hb-Difference | 0.700 | -1.354 - 2.754 | 0.472 |
| Ø3.8mm  8 Ports | Ø4.8mm  8 Ports | Incision-To-Suture Time | -4.259 | -17.394 - 8.876 | 0.517 |
