# Supplementary material for: Water-Jet Assisted Liposuction in Lipedema: Which Cannula is the Safest?
Source: Aesthet Surg J Open Forum. 2025 Sep 26;7:ojaf120. doi: 10.1093/asjof/ojaf120 (PMC12596102; doi:10.1093/asjof/ojaf120)
Supplement: ojaf120_Supplementary_Data [file ojaf120_supplementary_data.zip › sup_Table 2_1.docx]

Supplemental table 2: Patient demographics and disease characteristics grouped by the cannulas with the most ports used in the procedures. Percentages relate to number of cases, not the number of patients.

|  |  | 4 Ports | 8 Ports | p-Value |
| --- | --- | --- | --- | --- |
| Number of Cases |  | 179 | 64 |  |
| Stage - No. (%) | Stage I | 9 (5.0) | 0 (0.0) | 0.113 |
|  | Stage II | 73 (40.8) | 23 (35.9) |  |
|  | Stage III | 97 (54.2) | 41 (64.1) |  |
| Age in Years | Min | 20 | 19 |  |
|  | Average (SD) | 40 (12) | 42 (12) | 0.18 |
|  | Max | 70 | 70 |  |
| Weight in kg | Min | 62 | 62 |  |
|  | Average (SD) | 93.5 (18.9) | 94.2 (18.9) | 0.825 |
|  | Max | 159 | 147 |  |
|  | Missing Data - No. | 2 | 0 |  |
| BMI in kg/m2 | Min | 21.38 | 22.14 |  |
|  | Average (SD) | 32.55 (6.14) | 32.72 (5.52) | 0.844 |
|  | Max | 54.20 | 49.12 |  |
|  | Missing Data - No. | 2 | 0 |  |
| BMI by Stages - No. (%) | < 18.5 kg/m^2^ (Underweight) | 0 (0) | 0 (0) | 0.822 |
|  | 18.5-24.9 kg/m^2^ (Normal Weight) | 17 (9.6) | 6 (9.4) |  |
|  | 25.0-29.9 kg/m^2^ (Overweight) | 41 (23.2) | 12 (18.8) |  |
|  | 30.0- 34.9 kg/m^2^ (Obesity 1st Class) | 67 (37.9) | 27 (42.2) |  |
|  | 35.0-39.9 kg/m^2^ (Obesity 2nd Class) | 32 (18.1) | 14 (21.9) |  |
|  | > 40.0 kg/m^2^ (Extreme Obesity 3rd Class) | 20 (11.3) | 5 (7.8) |  |
| Diabetes Mellitus – No. (%) |  | 6 (3.4) | 3 (4.7) | 0.702 |
| Active Smokers |  | 33 (18.4) | 11 (17.2) | 1 |
