# Supplementary material for: Water-Jet Assisted Liposuction in Lipedema: Which Cannula is the Safest?
Source: Aesthet Surg J Open Forum. 2025 Sep 26;7:ojaf120. doi: 10.1093/asjof/ojaf120 (PMC12596102; doi:10.1093/asjof/ojaf120)
Supplement: ojaf120_Supplementary_Data [file ojaf120_supplementary_data.zip › sup_Table 3_1.docx]

Supplemental table 3: Patient demographics and disease characteristics for cases that only used the 3.8mm 4 ports or the 4.8mm 8 ports cannula. Percentages relate to number of cases, not number of patients.

|  |  | Ø 3.8mm 4 Ports | Ø 3.8mm 8 Ports | p-Value |
| --- | --- | --- | --- | --- |
| Number of Cases |  | 178 | 31 |  |
| Stage - No. (%) | Stage I | 9 (5.1) | 0 (0.0) | 0.228 |
|  | Stage II | 73 (41.0) | 10 (32.3) |  |
|  | Stage III | 96 (53.9) | 21 (67.7) |  |
| Age in Years | Min | 20 | 22 | 0.08 |
|  | Average (SD) | 40 (12) | 44 (12) |  |
|  | Max | 70 | 63 |  |
| Weight in kg | Min | 62 | 70 |  |
|  | Average (SD) | 93.6 (19.0) | 95.7 (16.8) | 0.56 |
|  | Max | 159 | 129 |  |
|  | Missing Data - No. | 2 | 0 |  |
| BMI in kg/m2 | Min | 21.38 | 23.66 |  |
|  | Average (SD) | 32.54 (6.15) | 33.162 (5.27) | 0.596 |
|  | Max | 54.20 | 43.20 |  |
|  | Missing Data - No. | 2 | 0 |  |
| BMI by Stages - No. (%) | < 18.5 kg/m^2^ (Underweight) | 0 (0.0) | 0 (0.0) | 0.837 |
|  | 18.5-24.9 kg/m^2^ (Normal Weight) | 17 (9.7) | 2 (6.5) |  |
|  | 25.0-29.9 kg/m^2^ (Overweight) | 41 (23.3) | 6 (19.4) |  |
|  | 30.0- 34.9 kg/m^2^ (Obesity 1st Class) | 67 (38.1) | 12 (38.7) |  |
|  | 35.0-39.9 kg/m^2^ (Obesity 2nd Class) | 31 (17.6) | 8 (25.8) |  |
|  | > 40.0 kg/m^2^ (Extreme Obesity 3rd Class) | 20 (11.4) | 3 (6.7) |  |
| Diabetes Mellitus – No. (%) |  | 6 (3.4) | 0 (0.0) | 0.595 |
| Active Smokers |  | 33 (18.5) | 4 (12.9) | 0.612 |
