# Supplementary material for: Water-Jet Assisted Liposuction in Lipedema: Which Cannula is the Safest?
Source: Aesthet Surg J Open Forum. 2025 Sep 26;7:ojaf120. doi: 10.1093/asjof/ojaf120 (PMC12596102; doi:10.1093/asjof/ojaf120)
Supplement: ojaf120_Supplementary_Data [file ojaf120_supplementary_data.zip › sup_Table 4_1_c.docx]

Supplemental table 4: Patient demographics and disease characteristics for cases that only used the 3.8mm 8 ports or the 4.8mm 8 ports cannula. Percentages relate to number of cases, not number of patients.

|  |  | 3.8mm 8 Ports | Ø 4.8mm 8 Ports | p-Value |
| --- | --- | --- | --- | --- |
| Number of Cases |  | 31 | 20 |  |
| Stage - No. (%) | Stage I | 0 (0.0) | 0 (0.0) | 0.154 |
|  | Stage II | 10 (32.3) | 11 (55.0) |  |
|  | Stage III | 21 (67.7) | 9 (45.0) |  |
| Age in Years | Min | 22 | 19 |  |
|  | Average (SD) | 44 (12) | 39 (11) | 0.126 |
|  | Max | 63 | 55 |  |
| Weight in kg | Min | 70 | 62 |  |
|  | Average (SD) | 95.7 (16.8) | 92.1 (22.5) | 0.572 |
|  | Max | 129 | 147 |  |
| BMI in kg/m2 | Min | 23.66 | 22.14 |  |
|  | Average (SD) | 33.16 (5.27) | 32.287 (6.55) | 0.720 |
|  | Max | 43.21 | 49.12 |  |
| BMI by Stages - No. (%) | < 18.5 kg/m^2^ (Underweight) | 0 (0.0) | 0 (0.0) |  |
|  | 18.5-24.9 kg/m^2^ (Normal Weight) | 2 (6.5) | 3 (15.0) | 0.315 |
|  | 25.0-29.9 kg/m^2^ (Overweight) | 6 (19.4) | 3 (15.0) |  |
|  | 30.0- 34.9 kg/m^2^ (Obesity 1st Class) | 12 (38.7) | 11 (55.0) |  |
|  | 35.0-39.9 kg/m^2^ (Obesity 2nd Class) | 8 (25.8) | 1 (5.0) |  |
|  | > 40.0 kg/m^2^ (Extreme Obesity 3rd Class) | 3 (6.7) | 2 (10.0) |  |
| Diabetes Mellitus – No. (%) |  | 0 (0.0) | 2 (10.0) | 0.155 |
| Active Smokers |  | 4 (12.9) | 6 (30.0) | 0.171 |
