# Supplementary material for: Water-Jet Assisted Liposuction in Lipedema: Which Cannula is the Safest?
Source: Aesthet Surg J Open Forum. 2025 Sep 26;7:ojaf120. doi: 10.1093/asjof/ojaf120 (PMC12596102; doi:10.1093/asjof/ojaf120)
Supplement: ojaf120_Supplementary_Data [file ojaf120_supplementary_data.zip › sup_Table 5_1.docx]

Supplemental table 5: Complications and procedure-related data grouped by the diameter of the biggest cannula. Percentages relate to number of cases, not number of patients.

|  |  | Ø 3.8mm | Ø 4.8mm | Number of Cases |
| --- | --- | --- | --- | --- |
| Number of Complications (%) | Perioperative Fluid Retentions | 47 (22.1) | 8 (26.7) | 243 |
|  | Infections | 18 (8.5) | 1 (3.3) | 243 |
|  | Necrosis of Skin | 4 (1.9) | 0 (0) | 243 |
|  | Blood Transfusions | 3 (1.4) | 0 (0) | 243 |
|  | Hematomas | 3 (1.4) | 0 (0) | 243 |
|  | Secondary Bleedings | 3 (1.4) | 0 (0) | 243 |
|  | Wound Healing Disorders | 1 (0.5) | 2 (6.7) | 243 |
|  | Uneven Skin | 1 (0.5) | 1 (3.3) | 243 |
| Aspirated Fat in ml | Min | 100 | 600 |  |
|  | Average (SD) | 3943.3 (1998.5) | 4953.1 (2607.1) |  |
|  | Max | 11100 | 11400 |  |
|  | Cases No. | 211 | 30 | 241 |
| Hemoglobin Difference in g/dl | Min | 0 | -2 |  |
|  | Average (SD) | -3.22 (1.72) | -4.02 (1.55) |  |
|  | Max | -7.4 | -7.1 |  |
|  | Cases No. | 59 | 9 | 68 |
| Hemoglobin Difference per 1000ml of Aspirated Fat in g/dl/1000ml |  | -0.8159 | -0.8103 |  |
| Incision-To-Suture Time in Minutes | Min | 25 | 35 |  |
|  | Average (SD) | 82.7 (27.3) | 77.3 (25.2) |  |
|  | Max | 186 | 139 |  |
|  | Cases No. | 209 | 30 | 239 |
| Incision-To-Suture Time per Liter Aspirated in min/1000ml of Aspirated Fat |  | 20.7963 | 15.5461 |  |
