# Supplementary material for: Water-Jet Assisted Liposuction in Lipedema: Which Cannula is the Safest?
Source: Aesthet Surg J Open Forum. 2025 Sep 26;7:ojaf120. doi: 10.1093/asjof/ojaf120 (PMC12596102; doi:10.1093/asjof/ojaf120)
Supplement: ojaf120_Supplementary_Data [file ojaf120_supplementary_data.zip › sup_Table 6_1.docx]

Supplemental table 6: Complications and procedure-related data grouped by the number of ports of the cannula with the most ports. Percentages relate to number of cases, not number of patients.

|  |  | 4 ports | 8 ports | Number of Cases |
| --- | --- | --- | --- | --- |
| Number of Complications (%) | Perioperative Fluid Retentions | 36 (20.1) | 19 (29.7) | 243 |
|  | Infections | 13 (7.3) | 58 (9.4) | 243 |
|  | Necrosis of Skin | 3 (1.7) | 1 (1.6) | 243 |
|  | Blood Transfusions | 2 (1.1) | 1 (1.6) | 243 |
|  | Hematomas | 3 (1.7) | 0 (0) | 243 |
|  | Secondary Bleedings | 1 (0.6) | 2 (3.1) | 243 |
|  | Wound Healing Disorders | 1 (0.6) | 2 (3.1) | 243 |
|  | Uneven Skin | 1 (0.6) | 1 (1.6) | 243 |
| Aspirated Fat in ml | Min | 100 | 600 |  |
|  | Average (SD) | 3733.9 (1979.2) | 5014.3 (2171.8) |  |
|  | Max | 11100 | 11400 |  |
|  | Cases No. | 178 | 63 | 241 |
| Hemoglobin Difference in g/dl | Min | 0 | -2.0 |  |
|  | Average (SD) | -2.98 (1.64) | -4.09 (1.61) |  |
|  | Max | -7.4 | -7.1 |  |
|  | Cases No. | 47 | 21 | 68 |
| Hemoglobin Difference per 1000ml of Aspirated Fat in g/dl/1000ml |  | -0.7977 | -0.8159 |  |
| Incision-To-Suture Time in Minutes | Min | 25 | 35 |  |
|  | Average (SD) | 83.6 (27.3) | 74.7 (23.6) |  |
|  | Max | 186 | 147 |  |
|  | Cases No. | 176 | 63 | 239 |
| Incision-To-Suture Time per Liter Aspirated in min/1000ml of Aspirated Fat |  | 22.4959 | 14.9581 |  |
