# Supplementary material for: Water-Jet Assisted Liposuction in Lipedema: Which Cannula is the Safest?
Source: Aesthet Surg J Open Forum. 2025 Sep 26;7:ojaf120. doi: 10.1093/asjof/ojaf120 (PMC12596102; doi:10.1093/asjof/ojaf120)
Supplement: ojaf120_Supplementary_Data [file ojaf120_supplementary_data.zip › sup_Table 7_1.docx]

Supplemental table 7: Complications and procedure-related data for procedures that only used the 3.8mm 4 ports or only the 3.8mm 8 ports cannula. Percentages relate to number of cases, not number of patients.

|  |  | Ø 3.8mm 4 ports | Ø 3.8mm 8 ports | Number of Cases |
| --- | --- | --- | --- | --- |
| Number of Complications (%) | Perioperative Fluid Retentions | 36 (20.2) | 11 (35.5) | 209 |
|  | Infections | 13 (7.3) | 5 (16.1) | 209 |
|  | Necrosis of Skin | 3 (1.7) | 1 (3.2) | 209 |
|  | Blood Transfusions | 2 (1.1) | 1 (3.2) | 209 |
|  | Hematomas | 3 (1.7) | 0 (0) | 209 |
|  | Secondary Bleedings | 1 (0.6) | 2 (6.5) | 209 |
|  | Wound Healing Disorders | 1 (0.6) | 0 (0) | 209 |
|  | Uneven Skin | 0 (0) | 0 (0) | 209 |
| Aspirated Fat in ml | Min | 100 | 2700 |  |
|  | Average (SD) | 3738.0 (1984.5) | 5247.0 (1671.0) |  |
|  | Max | 11100 | 8200 |  |
|  | Cases No. | 177 | 30 | 207 |
| Hemoglobin Difference in g/dl | Min | 0 | -2.0 |  |
|  | Average (SD) | -2.98 (1.66) | -4.35 (1.48) |  |
|  | Max | -7.4 | -7.0 |  |
|  | Cases No. | 47 | 11 | 58 |
| Hemoglobin Difference per 1000ml of Aspirated Fat in g/dl/1000ml |  | -0.7969 | -0.8282 |  |
| Incision-To-Suture Time in Minutes | Min | 25 | 39 |  |
|  | Average (SD) | 84.5 (27.4) | 70.1 (19.6) |  |
|  | Max | 186 | 124 |  |
|  | Cases No. | 175 | 30 | 205 |
| Incision-To-Suture Time per Liter Aspirated in min/1000ml of Aspirated Fat |  | 22.4719 | 13.3409 |  |
