# Supplementary material for: Water-Jet Assisted Liposuction in Lipedema: Which Cannula is the Safest?
Source: Aesthet Surg J Open Forum. 2025 Sep 26;7:ojaf120. doi: 10.1093/asjof/ojaf120 (PMC12596102; doi:10.1093/asjof/ojaf120)
Supplement: ojaf120_Supplementary_Data [file ojaf120_supplementary_data.zip › sup_Table 8_1_c.docx]

Supplemental table 8: Complications and procedure-related data for procedures that only used the 3.8mm 8 ports or only the 4.8mm 8 ports cannula. Percentages relate to number of cases, not number of patients.

|  |  | 3.8mm 8 ports | Ø 4.8mm 8 ports | Number of Cases |
| --- | --- | --- | --- | --- |
| Number of Complications (%) | Perioperative Fluid Retentions | 11 (35.5) | 5 (25.0) | 51 |
|  | Infections | 5 (16.1) | 1 (5.0) | 51 |
|  | Necrosis of Skin | 1 (3.2) | 0 (0.0) | 51 |
|  | Blood Transfusions | 1 (3.2) | 0 (0.0) | 51 |
|  | Hematomas | 0 (0) | 0 (0.0) | 51 |
|  | Secondary Bleedings | 2 (6.5) | 0 (0.0) | 51 |
|  | Wound Healing Disorders | 0 (0) | 1 (5.0) | 51 |
|  | Uneven Skin | 0 (0) | 1 (5.0) | 51 |
| Aspirated Fat in ml | Min | 2700 | 600 |  |
|  | Average (SD) | 5247.0 (1671.0) | 4875.0 (2847.8) |  |
|  | Max | 8200 | 11400 |  |
|  | Cases No. | 30 | 20 | 50 |
| Hemoglobin Difference in g/dl | Min | -2.0 | -2.6 |  |
|  | Average (SD) | -4.35 (1.48) | -4.78 (1.89) |  |
|  | Max | -7.0 | -7.1 |  |
|  | Cases No. | 11 | 4 | 15 |
| Hemoglobin Difference per 1000ml of Aspirated Fat in g/dl/1000ml |  | -0.8282 | -0.9805 |  |
| Incision-To-Suture Time in Minutes | Min | 39 | 35 |  |
|  | Average (SD) | 70.1 (19.6) | 74.0 (27.9) |  |
|  | Max | 124 | 139 |  |
|  | Cases No. | 30 | 20 | 50 |
| Incision-To-Suture Time per Liter Aspirated in min/1000ml of Aspirated Fat |  | 13.3409 | 15.1794 |  |
