# Supplementary material for: Water-Jet Assisted Liposuction in Lipedema: Which Cannula is the Safest?
Source: Aesthet Surg J Open Forum. 2025 Sep 26;7:ojaf120. doi: 10.1093/asjof/ojaf120 (PMC12596102; doi:10.1093/asjof/ojaf120)
Supplement: ojaf120_Supplementary_Data [file ojaf120_supplementary_data.zip › sup_Table 9_1.docx]

Supplemental table 9: Statistical test to find significant differences between the groups.

| Group 1 | Group 2 | Complication | Odds Ratio/Mean Difference | 95% Confidence Interval | p-Value |
| --- | --- | --- | --- | --- | --- |
| Ø 3.8mm | Ø 4.8mm | Perioperative Fluid Retentions | 1.284 | 0.537 - 3.070 | 0.641 |
| Ø 3.8mm | Ø 4.8mm | Infections | 0.374 | 0.048 – 2.905 | 0.482 |
| Ø 3.8mm | Ø 4.8mm | Necrosis of Skin | - | - | 1.000 |
| Ø 3.8mm | Ø 4.8mm | Blood Transfusions | - | - | 1.000 |
| Ø 3.8mm | Ø 4.8mm | Hematomas | - | - | 1.000 |
| Ø 3.8mm | Ø 4.8mm | Secondary Bleedings | - | - | 1.000 |
| Ø 3.8mm | Ø 4.8mm | Wound Healing Disorders | 15.143 | 1.330 – 172,452 | **0.041** |
| Ø 3.8mm | Ø 4.8mm | Uneven Skin | 7.310 | 0.445 – 120.077 | 0.232 |
| Ø 3.8mm | Ø 4.8mm | Aspirated Fat Volume | -1010.442 | -1810.414 -  -210.471 | **0.014** |
| Ø 3.8mm | Ø 4.8mm | Hb-Difference | 0.796 | -0.4092 – 2.002 | 0.192 |
| Ø 3.8mm | Ø 4.8mm | Incision-To-Suture Time | 4.509 | -5.747 – 14.765 | 0.387 |
| 4 Ports | 8 Ports | Perioperative Fluid Retentions | 1.677 | 0.876 – 3.209 | 0.121 |
| 4 Ports | 8 Ports | Infections | 1.321 | 0.480 -3.636 | 0.593 |
| 4 Ports | 8 Ports | Necrosis of Skin | 0.931 | 0.095 – 9.117 | 1.000 |
| 4 Ports | 8 Ports | Blood Transfusions | 1.405 | 0.125 – 15.760 | 1.000 |
| 4 Ports | 8 Ports | Hematomas | - | - | 0.568 |
| 4 Ports | 8 Ports | Secondary Bleedings | 5.742 | 0.512 – 64.431 | 0.170 |
| 4 Ports | 8 Ports | Wound Healing Disorders | 5.742 | 0.512 – 64.431 | 0.170 |
| 4 Ports | 8 Ports | Uneven Skin | -1280.297 | -1866.795 –  -693.799 | **<0.001** |
| 4 Ports | 8 Ports | Aspirated Fat Volume | 2.825 | 0.174 – 45.847 | 0.458 |
| 4 Ports | 8 Ports | Hb-Difference | 1.113 | 0.260 – 1.966 | **0.011** |
| 4 Ports | 8 Ports | Incision-To-Suture Time | 8.889 | 1.249 – 16.530 | **0.023** |
